# Significance of molecular diagnostics for therapeutic decision-making in recurrent glioma

**DOI:** 10.1093/noajnl/vdad060

**Published:** 2023-05-12

**Authors:** Jens Blobner, Laura Dengler, Sven Blobner, Constantin Eberle, Jonathan Weller, Nico Teske, Philipp Karschnia, Katharina Rühlmann, Kathrin Heinrich, Frank Ziemann, Philipp A Greif, Irmela Jeremias, Rachel Wuerstlein, Korbinian Hasselmann, Mario Dorostkar, Patrick N Harter, Stefanie Quach, Veit Stoecklein, Nathalie L Albert, Maximilian Niyazi, Joerg-Christian Tonn, Niklas Thon, Benedikt Christoph Westphalen, Louisa von Baumgarten

**Affiliations:** Department of Neurosurgery, LMU University Hospital, Ludwig Maximilians University (LMU), Munich, Germany; German Cancer Consortium (DKTK), Partner Site Munich, Germany; Department of Neurosurgery, LMU University Hospital, Ludwig Maximilians University (LMU), Munich, Germany; German Cancer Consortium (DKTK), Partner Site Munich, Germany; Medical Faculty Heidelberg, University of Heidelberg, Heidelburg, Germany; Department of Neurosurgery, LMU University Hospital, Ludwig Maximilians University (LMU), Munich, Germany; German Cancer Consortium (DKTK), Partner Site Munich, Germany; Department of Neurosurgery, LMU University Hospital, Ludwig Maximilians University (LMU), Munich, Germany; German Cancer Consortium (DKTK), Partner Site Munich, Germany; Department of Neurosurgery, LMU University Hospital, Ludwig Maximilians University (LMU), Munich, Germany; German Cancer Consortium (DKTK), Partner Site Munich, Germany; Department of Neurosurgery, LMU University Hospital, Ludwig Maximilians University (LMU), Munich, Germany; German Cancer Consortium (DKTK), Partner Site Munich, Germany; Comprehensive Cancer Center München (CCC München), LMU University Hospital, Ludwig Maximilians University (LMU), Munich, Germany; Department of Medicine, Hematology and Oncology Division and Cellular Immunotherapy Program, LMU University Hospital, Ludwig Maximilians University (LMU), Munich, Germany; Department of Medicine, Hematology and Oncology Division and Cellular Immunotherapy Program, LMU University Hospital, Ludwig Maximilians University (LMU), Munich, Germany; Department of Medicine, Hematology and Oncology Division and Cellular Immunotherapy Program, LMU University Hospital, Ludwig Maximilians University (LMU), Munich, Germany; German Cancer Consortium (DKTK), Partner Site Munich, Germany; Dr. von Haunersches Children Hospital, LMU University Hospital, Ludwig Maximilians University (LMU), Munich, Germany; Department of Obstetrics and Gynecology and CCC Munich LMU University Hospital, Ludwig Maximilians University (LMU), Munich, Germany; German Cancer Consortium (DKTK), Partner Site Munich, Germany; Department of Medicine, Hematology and Oncology Division and Cellular Immunotherapy Program, LMU University Hospital, Ludwig Maximilians University (LMU), Munich, Germany; Comprehensive Cancer Center München (CCC München), LMU University Hospital, Ludwig Maximilians University (LMU), Munich, Germany; German Cancer Consortium (DKTK), Partner Site Munich, Germany; Comprehensive Cancer Center München (CCC München), LMU University Hospital, Ludwig Maximilians University (LMU), Munich, Germany; Center for Neuropathology and Prion Research, LMU University Hospital, Ludwig Maximilians University (LMU), Munich, Germany; German Cancer Consortium (DKTK), Partner Site Munich, Germany; Comprehensive Cancer Center München (CCC München), LMU University Hospital, Ludwig Maximilians University (LMU), Munich, Germany; Bavarian Cancer Research Center (BZKF), Erlangen, Germany; Department of Neurosurgery, LMU University Hospital, Ludwig Maximilians University (LMU), Munich, Germany; German Cancer Consortium (DKTK), Partner Site Munich, Germany; Department of Neurosurgery, LMU University Hospital, Ludwig Maximilians University (LMU), Munich, Germany; German Cancer Consortium (DKTK), Partner Site Munich, Germany; Department of Nuclear Medicine, LMU University Hospital, Ludwig Maximilians University (LMU), Munich, Germany; Bavarian Cancer Research Center (BZKF), Erlangen, Germany; Department of Radiation Oncology, LMU University Hospital, Ludwig Maximilians University (LMU), Munich, Germany; Department of Neurosurgery, LMU University Hospital, Ludwig Maximilians University (LMU), Munich, Germany; German Cancer Consortium (DKTK), Partner Site Munich, Germany; Department of Neurosurgery, LMU University Hospital, Ludwig Maximilians University (LMU), Munich, Germany; German Cancer Consortium (DKTK), Partner Site Munich, Germany; German Cancer Consortium (DKTK), Partner Site Munich, Germany; Department of Medicine, Hematology and Oncology Division and Cellular Immunotherapy Program, LMU University Hospital, Ludwig Maximilians University (LMU), Munich, Germany; Comprehensive Cancer Center München (CCC München), LMU University Hospital, Ludwig Maximilians University (LMU), Munich, Germany; Bavarian Cancer Research Center (BZKF), Erlangen, Germany; Department of Neurosurgery, LMU University Hospital, Ludwig Maximilians University (LMU), Munich, Germany; German Cancer Consortium (DKTK), Partner Site Munich, Germany; Department of Neurology, LMU University Hospital, Ludwig Maximilians University (LMU), Munich, Germany; Bavarian Cancer Research Center (BZKF), Erlangen, Germany

**Keywords:** molecular-matched targeted therapy, molecular tumor board, recurrent glioma

## Abstract

**Background:**

Targeted therapies have substantially improved survival in cancer patients with malignancies outside the brain. Whether in-depth analysis for molecular alterations may also offer therapeutic avenues in primary brain tumors remains unclear. We herein present our institutional experience for glioma patients discussed in our interdisciplinary *molecular tumor board* (MTB) implemented at the Comprehensive Cancer Center Munich (LMU).

**Methods:**

We retrospectively searched the database of the MTB for all recurrent glioma patients after previous therapy. Recommendations were based on next-generation sequencing results of individual patient’s tumor tissue. Clinical and molecular information, previous therapy regimens, and outcome parameters were collected.

**Results:**

Overall, 73 consecutive recurrent glioma patients were identified. In the median, advanced molecular testing was initiated with the third tumor recurrence. The median turnaround time between initiation of molecular profiling and MTB case discussion was 48 ± 75 days (range: 32–536 days). Targetable mutations were found for 50 recurrent glioma patients (68.5%). IDH1 mutation (27/73; 37%), epidermal growth factor receptor amplification (19/73; 26%), and NF1 mutation (8/73; 11%) were the most detected alterations and a molecular-based treatment recommendation could be made for all of them. Therapeutic recommendations were implemented in 12 cases (24%) and one-third of these heavily pretreated patients experienced clinical benefit with at least disease stabilization.

**Conclusions:**

In-depth molecular analysis of tumor tissue may guide targeted therapy also in brain tumor patients and considerable antitumor effects might be observed in selected cases. However, future studies to corroborate our results are needed.

Key Points

1. In-depth molecular analysis to detect clinically relevant molecular somatic alterations is feasible in recurrent glioma patients.
2. Molecularly matched targeted therapy for glioma patients is well tolerated and might be associated with a clinical benefit in a subset of patients.

**Importance of the Study**

Tremendous progress was made in treating oncologic patients with malignancies outside of the brain. However, even though huge efforts are made within the field of Neuro-oncology, prognosis of patients with recurrent malignant glioma remains dismal. Targeted therapies based on sequencing analysis of patients’ individual tumors improved survival of many cancer patients. Therefore, we retrospectively investigated whether in-depth analysis for molecular alterations may also offer therapeutic avenues for brain tumor patients. Overall, 73 consecutive recurrent glioma patients were identified and targetable mutations were found in 68.5%. For all these patients a molecular-based treatment recommendation could be made and more than one-third of these heavily pretreated patients showed clinical benefit with at least disease stabilization. Even though considerable antitumor effects might be observed in selected cases further studies are needed to corroborate our results.

Gliomas are often characterized by their infiltrative growth, and standard of care treatment still rests on microsurgical resection often followed by radio- and chemotherapy.^1^ However, neither aggressive surgery nor radiotherapeutic or medical approaches represent curative approaches and progression inevitably occurs.^2,3^ Although extensive research efforts have been undertaken to identify viable therapeutic vulnerabilities, effective treatment options in patients relapsing after first-line therapy are scarce and prognosis at recurrence, especially for high-grade glioma, is often dismal.^4–6^ Therefore, novel therapeutic strategies are urgently needed.

The use of molecularly matched targeted therapies has been shown to be a feasible and efficacious way to treat selected patients with various systemic tumor entities.^7^ Furthermore, preliminary data suggests that also across different tumor entities like melanoma or lung cancer, molecularly guided treatment approaches may be superior to unmatched empiric therapy.^8^ Recently, the FDA has issued tumor agnostic approval for selected targeted therapies.^9^ However, there is an ongoing controversy as to what extend the cellular context as well as the tumor microenvironment may remain important for the vast majority of genomic variants.^10^ Furthermore, conventional matching of patients and treatments based on the individual genomic profile is supposed to be beneficial under the prerequisite that the respective molecular alteration is principally present in cancer cells and maintained at a stable level within the tumor during the course of disease. Additionally, the interpretation of the relationship between individual molecular alterations and the particular clinical outcome is further complicated by the presence of potentially interfering mutations. This may be of specific relevance for brain tumors like glioma, as the latter are characterized by a pronounced genetic instability, inter- and intratumoral heterogeneity, and a highly pro-tumorigenic microenvironment. Furthermore, the biology of brain tumors is represented by a complex interaction of genetic and non-genetic mechanisms. Consequently, sequencing approaches alone may lead to an underestimation of the pathological behavior.^11^

Therefore, the therapeutic value of molecularly matched therapies for the majority of known driver mutations as well as variants with unknown significance for glioma patients remains to be determined. The evaluation of personalized treatment strategies in this heterogeneous patient cohort is challenging and current evidence for their feasibility and efficacy is scarce.^12–14^ However, due to a lack of therapeutic options, molecularly matched off-label treatments are considered after discussion in molecular tumor boards for patients with relapsing glioma in a good condition in many centers across Europe and even reimbursement can be granted by health insurances.^15,16^

Since little information on the outcome of molecularly targeted therapies in glioma patients are available, we, therefore, determined its feasibility and potential relevance in a retrospective analysis of 73 consecutive recurrent glioma patients who received comprehensive molecular analysis and subsequent evaluation in our molecular tumor board (MTB).

## Material and Methods

### Study Population

We searched the institutional database of the Comprehensive Cancer Center of the Medical Faculty of the Ludwig Maximilians University for patients with recurrent glioma who were discussed within the MTB between January 2020 and June 2021. We collected demographic information and clinical presentation; histopathology; treatment specifics and clinical outcome. This study was approved by the local ethics committee of the Ludwig Maximilians University (application number: 21-0869).

### Molecular Tumor Board

Decision for next-generation sequencing (NGS) analysis is made within the framework of an interdisciplinary neuro-oncology tumor board per clinical judgment of necessity. Main criteria were progressive disease (PD) without reasonable standard of care treatment options in patients with a good clinical condition and suspected life expectancy >3 months, an unusual clinical course, or diagnostic uncertainty. Whenever possible, NGS was performed using tissue samples that were collected after the last line of treatment. All patients receiving NGS analysis were discussed in the interdisciplinary MTB regardless of the current treatment status and molecular alterations are considered as relevant if their variant allele frequency exceeds 5%.

MTB in our center comprises neuro-oncologists, neuropathologists, pathologists, hemato-oncologists, and human geneticists. Clinical history and molecular profile of each patient are reviewed and the potential actionability of the discovered mutations, as well as the data for blood-brain-barrier permeability, is discussed by reviewing literature and publicly available databases, such as PubMED, clinicaltrials.gov, ClinVar, Varsome, OncoKB, and CIViC.^17^ Treatment recommendations are supported by levels of evidence for molecular targets by using the European Society for Medical Oncology (ESMO) Scale for Clinical Actionability of Molecular Targets (ESCAT). The ESCAT scale defines 6 levels of evidence for molecular targets in order to offer a common language in cancer medicine to prioritize genomic alterations as markers to select patients for targeted therapies.^18^ Therapeutic recommendations of the MTB are implemented if clinically indicated (recurrent or PD and no approved therapeutic alternatives) after discussion in the neuro-oncologic tumor board. As most targeted treatment options have not been officially authorized for brain tumor patients, approval for reimbursement by health insurance was requested. Follow-up MRI- scans under therapy were performed every 8–12 weeks.

### Molecular Pathology

Molecular analysis was performed by the Center for Neuropathology of the Ludwig Maximilians University Munich. For sequencing, fresh frozen and formalin-fixed paraffin-embedded tissue was used. Targeted NGS sequencing was performed using the TruSight Oncology 500 assay targeting 523 genes for the assessment of DNA and RNA variant types including single nucleotide variants and insertions/deletions (indels) as well as copy number variations, gene fusions, microsatellite instability (MSI) status and Tumor mutational burden (TMB) (mutations per megabase) (TMB). TMB was classified as high if the mutational load exceeds 10 mutations/megabase.

### Analysis of the Results

In order to determine the clinical impact of panel-guided NGS-adjusted therapies, we compared the progression-free survival (PFS) with the PFS of the latest treatment modality defined as the interval from therapy onset until progression or tumor recurrence on the basis of MRI-defined recurrence according to the updated criteria for response assessment for glioma.^19^

### Data Assessment and Statistical Analysis

All statistical analyses were performed using Prism statistical software (Prism 9.3.1(350); GraphPad Software, LLC., San Diego, CA, USA). The significance level was set at *P* ≤ .05. All values are expressed as mean ± standard error of the mean if not indicated otherwise, and range is given.

## Results

### Characteristics of Neuro-Oncologic Patients

**Figure 1.**
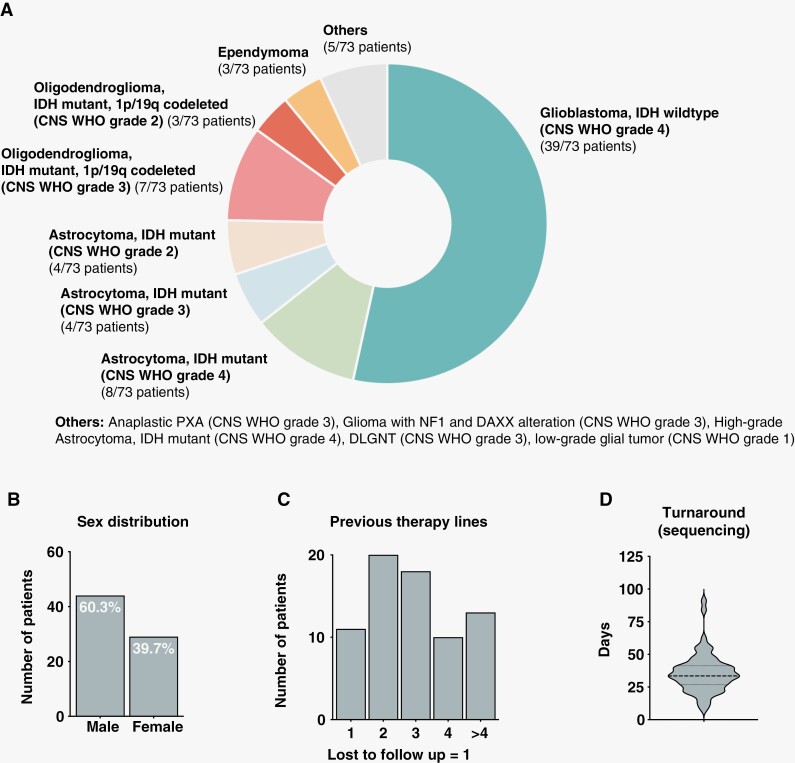
Patient characteristics. **A** Distribution of the respective subclassifications of recurrent glioma patients (*n* = 73) discussed in the molecular tumor board (MTB) between January 2020 and June 2021 (reclassified according to WHO 2021). **B** Sex distribution of *n* = 73 recurrent glioma patients. **C** Number of previous therapy lines (surgery, chemotherapy, or radiation) of (*n* = 73) recurrent glioma patients. The majority of the patients were discussed within the framework of the MTB after at least 3 treatment modalities. **D** Turnaround time for next-generation sequencing diagnostic.

From January 2020 until June 2021, 73 patients with recurrent or progressive glioma were discussed in our MTB. 60.2% (*n* = 44) were male (median age 52; range 18–77 years) and 39.8% (*n* = 29) were female (median age 50; range 26–70 years). The most common tumors Glioblastoma, IDH-wild-type (CNS WHO grade 4) (39/73; 53.4%), followed by Astrocytoma, IDH mutant (CNS WHO grade 4) (8/73; 11%), Oligodendroglioma, IDH mutant and 1p/19q-codeleted (CNS WHO grade 3) (7/73; 9.6%), Astrocytoma, IDH mutant (CNS WHO grade 3) (4/73; 5.5%), Astrocytoma, IDH mutant (CNS WHO grade 2) (4/73; 5.5%), Oligodendroglioma, IDH mutant and 1p/19q-codeleted (CNS WHO grade 2) (3/73; 4.1%) and ependymoma (3/73; 4.1%) (Figure 1A+B). The majority were heavily pretreated with at least 3 completed treatment modalities prior to MTB discussion (Figure 1C).

### Molecular Profiling and Treatment Recommendations

NGS was performed for all 73 patients with recurrent glioma and the median number of therapy lines before advanced molecular testing was initiated was 3 (range: 1–10) (Figure 1C). We attempted to ensure that recurrent tumor tissue was used for molecular profiling in order to provide the most accurate analysis of sequencing data, which was successful in 95% (69/73) of our cases. Furthermore, treatment according to those alterations was implemented, whenever possible, as the next line of treatment. The median turnaround time for completion of molecular profiling was 34 ± 15 days (range: 8–91 days) (Figure 1D, Supplementary Figure 1). The median turnaround time between initiation of molecular profiling and MTB case discussion was 48 ± 75 days (range: 32–536 days) (Supplementary Figure 1**).** NGS sequencing was successful in 98.6% (72/73). In one case insufficient material quality results in technically unsuccessful molecular analysis.

**Figure 2.**
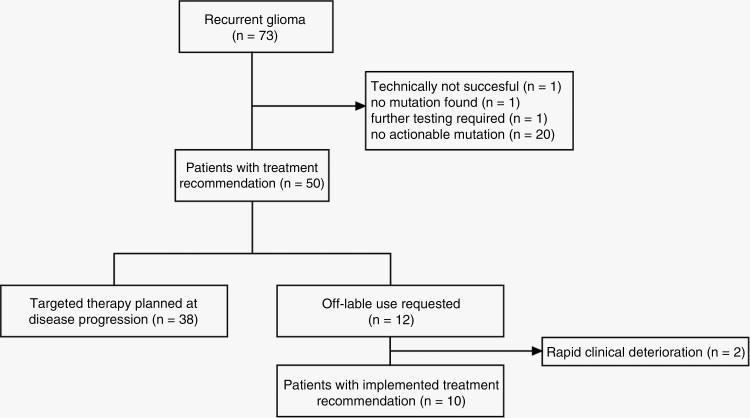
Therapeutic management of glioma patients within the molecular tumor board between January 2020 and June 2021. Flow chart indicating the yield of actionable mutations in 73 patients with recurrent glioma. Out of 50 patients with a treatment recommendation off-label use was requested in 12 of these cases. Due to clinical deterioration the therapeutic objective was changed for 2 patients. In 2 cases, cost coverage from health insurance was given but targeted therapy was initiated after the deadline of data collection.

**Figure 3.**
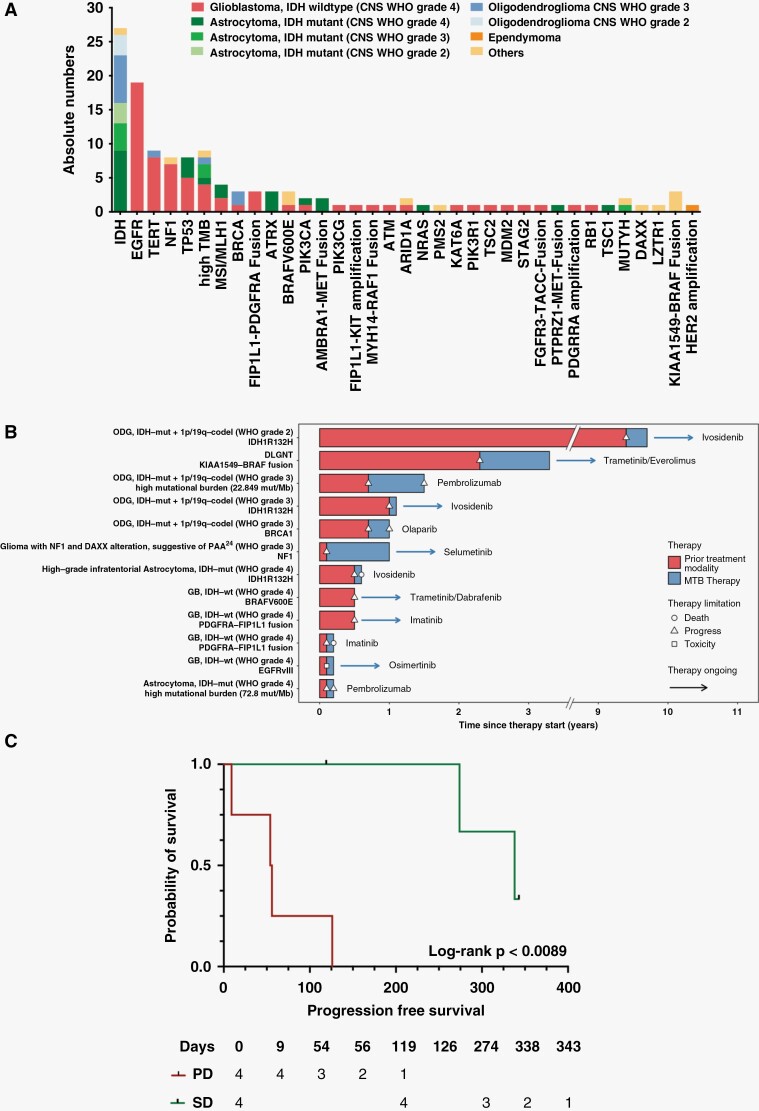
Clinical significance of a molecular-based targeted therapy. **A** Frequency of genomic alterations in *n* = 73 patients with recurrent glioma**. B** Swimmer plot depicting the clinical course of glioma patients under conventional and molecular-matched treatment regimens. Horizontal bars illustrate treatment duration as well as response and disease progression of respective therapies. **C** Progression free survival of glioma patients with at least stable disease or progressive disease at first follow-up MRI scan after initiation of molecular tumor board treatment recommendation.

Overall, actionable mutations, as classified by the MTB, were found in 50/73 patients (68.5%). Molecular alterations are considered as actionable if potential targeted therapies are available. For one patient no mutation could be found at all and for 20/73 patients (27.4%) the obtained molecular alterations were not targetable. In one patient with epidermal growth factor receptor mutation, further testing for preserved phosphatase and tensin homolog gene (PTEN) expression was required before a treatment recommendation could be made (Figure 2). In total, we discovered 172 molecular alterations in 35 different genes. IDH1 mutation was the most common alteration (37%; 27/73 patients), followed by epidermal growth factor receptor (26%; 19/73 patients) and NF1 (11%; 8/73 patients) alterations. TMB was high in 9.6% (7/73 patients). Frequency of molecular alterations and distribution by tumor type are shown in Figure 3A. For all 50 patients with targetable mutations, a therapeutic recommendation was issued, while 38 patients (76%) are still under conventional treatment options with planned implementation at disease progression.

For all patients with implemented treatment recommendations (12/50; 24%), reimbursement of targeted therapy by the health insurance was requested and approved after 27 days in the median. However, due to the time between acquisition of NGS results (mean 34 days ± 15), interdisciplinary discussion (mean 48 days ± 75), application for health-insurance reimbursement (27 days ± 22) and start of the therapy a relevant number of patients experienced progression or clinical deterioration (2/12; 16.7%) (Figure 2).

### Clinical Outcome

Treatment duration and clinical course of patients with recurrent glioma under molecular-matched targeted therapy is illustrated in Figure 3B. Follow-up information was available for all patients with a treatment recommendation until the end of the data collection period. Out of 12 patients following the MTB recommendation, 6 patients (6/12; 50%) showed durable response with at least disease stabilization, and 25% (3/12) showed a longer PFS compared to the previous treatment modality (Figure 3B; Table 1). On follow-up MRI after initiation of a targeted therapy, 33.3% (4/12) of the cases showed PD, 33.3% (4/12) showed at least stable disease and in 33.3% (4/12) the therapy was recently started and follow-up MRI scans were planned after the end of data collection. For all patients, no case of regimen limiting toxicity was observed. According to Luger et al. 2 groups were defined: One group comprising all patients with at least SD at first follow-up MRI scan and another group with PD at first MRI.^15^ Median PFS of patients with at least SD was 338 days (range 119–343 days) and 55 days (range 9–126 days) for patients with PD at first follow-up (*P* < .01) (Figure 3C). However, the cohort with PD at first MRI scan comprises a higher proportion of CNS WHO grade 4 tumors (1 patient in SD group, 3 patients in PD group). As the assessment of clinical utility in patients at various stages of their illness is challenging, we calculated the PFS ratio as an intra-individual outcome parameter as recently described by Mock et al. and widely used in current precision oncology trials.^20–22^ In this setting, each patient serves as his/her own control thereby circumventing the need for a control arm. As cancer dynamic is accelerating during course of disease a PFS ratio >1.3 is considered as a surrogate parameter of response. We detected PFS ratio >1.3 in 25% (2/8) of our evaluable patients which is in line with other studies investigating targeted therapies in CNS- and non-CNS tumors (Table 2, Supplementary Figure 2).^21,23^

**Table 1.** Characteristics and Medical History of Patients With Implemented Molecular Tumor Board Treatment Recommendation

Demographics			Histology		Previous Treatment Lines								
Age	Sex	Karnofsky Performance score (KPS)	Diagnosis	MGMT Promoter Methylated	First	Second	Third	Forth	Fifth	Sixth	Seventh	Last Treatment Modality	RLT
34	female	80	Oligdendroglioma, IDH-mutant and 1p/19q-codeleted (CNS WHO Grade 3)	yes	TMZ	Surgery	PCV	RT	BT	Surgery	RT	Radiation	no
43	female	40	Glioblastoma, IDH-wild-type (CNS WHO Grade 4)	no	Surgery	RCx	TMZ					TMZ	no
33	male	90	Astrocytoma, IDH-mutant (CNS WHO Grade 4)	yes	Surgery	RCx	RT	TMZ /CCNU				CCNU	no
47	male	70	Glioma with NF1 and DAXX alteration, suggestive of PAA (CNS WHO Grade 3)^24^	no	Surgery	RCx	TMZ	CCNU				CCNU	no
47	male	80	Diffuse leptomeningeal glioneural tumor	no	Surgery	RT	Surgery	RT				Re-Irradiation	no
29	male	100	Oligdendroglioma, IDH-mutant and 1p/19q-codeleted (CNS WHO Grade 3)	yes	Surgery	TMZ	RT	BT	Surgery	PC	RT	Re-Irradiation	no
56	female	60	Oligdendroglioma, IDH-mutant and 1p/19q-codeleted (CNS WHO Grade 2)	yes	Surgery	RCx	TMZ	RT				Re-Irradiation	no
24	male	60	Glioblastoma, IDH-wild-type (CNS WHO Grade 4)	no	Surgery	Surgery	RT	Surgery				Surgery	no
57	male	60	Glioblastoma, IDH-wild-type (CNS WHO Grade 4)	yes	Surgery	RCx	TMZ /CCNU					TMZ + CCNU (CeTeG)	no
52	female	70	Oligdendroglioma, IDH-mutant and 1p/19q-codeleted (CNS WHO Grade 3)	yes	Surgery	RCx	PC					Procarbazine /Lomustine	no
18	male	90	High-grade infratentorial Astrozytoma, IDH-mutant (CNS WHO Grade 4)	no	RCx	RT	CPI					Nivolumab	no
47	female	80	Glioblastoma, IDH-wild-type (CNS WHO Grade 4)	no	RCx	TMZ + TTF						TMZ + TTF	yes

**Table 2.** Molecular Tumor Board Recommendation Implementation and Outcome Measurement of Molecular-Based Targeted Therapy.

Diagnosis	Molecular Diagnostic				Outcome Parameters								
	Tissue Specimen for Next-Generation Sequencing	Turnnaround Sequencing	Timeframe (Tissue–Discussion)	Target	Last Treatment Modality	PFS1	Implemented Therapy	ESCAT	Reimbursement	Steroids at Baseline	PFS2	Ongoing	PFS Ratio
Oligdendroglioma, IDH-mutant and 1p/19q-codeleted (CNS WHO Grade 3)	resection	49	62	High mutational burden (22,849 mut. per megabase)	Radiation	263	Pembrolizumab	IIB	14	no	274	no	1.04
Glioblastoma, IDH-wild-type (CNS WHO Grade 4)	biopsy	53	85	PDGFRA-FIP1L1 Fusion	TMZ	27	Imatinib	IIIA	4	ja	9	no	0.33
Astrocytoma, IDH-mutant (CNS WHO Grade 4)	resection	41	128	High mutational burden (72,8 mut. per megabase)	CCNU	42	Pembrolizumab	IIB	15	no	56	no	1.33
Glioma with NF1 and DAXX alteration, suggestive of PAA (CNS WHO Grade 3)^24^	biopsy	28	164	NF1	CCNU	54	Selumetinib	IIIA	88	no	343	yes	6.35
Diffuse leptomeningeal glioneural tumor	resection	27	2701	KIAA1549-BRAF Fusion	Re-Irradiation	839	Trametinib /Everolimus	IID	33	no	338	yes	0.40
Oligdendroglioma, IDH-mutant and 1p/19q-codeleted (CNS WHO Grade 3)	resection	55	754	BRCA1 + IDH2	Re-Irradiation	246	Olaparib	IIA	32	no	126	no	0.51
Oligdendroglioma, IDH-mutant and 1p/19q-codeleted (CNS WHO Grade 2)	biopsy	28	47	IDHR132H	Re-Irradiation	3423	Ivosidenib	IIA	41	no	119	yes	0.03
Glioblastoma, IDH-wild-type (CNS WHO Grade 4)	resection	25	80	PDGFRA-FIP1L1 Fusion	Surgery	194	Imatinib	IIIA	lost to follow up				
Glioblastoma, IDH-wild-type (CNS WHO Grade 4)	resection	34	158	BRAFV600E	TMZ + CCNU (CeTeG)	168	Dabrafenib /Trametinib	IIA	20	yes	---	---	---
Oligdendroglioma, IDH-mutant and 1p/19q-codeleted (CNS WHO Grade 3)	biopsy	39	56	IDHR132H	Procarbazine /Lomustine	359	Ivosidenib	IIA	33	yes	45	yes	0.13
High-grade infratentorial Astrozytoma, IDH-mutant (CNS WHO Grade 4)	biopsy	39	52	IDHR132H	Nivolumab	179	Ivosidenib	IIA	27	no	54	no	0.30
Glioblastoma, IDH-wild-type (CNS WHO Grade 4)	biopsy	15	112	EGFR mutation	TMZ + TTF	49	Osimertinib	IIIA	21	yes	16	yes	0.33

### Case Example

**Figure 4.**
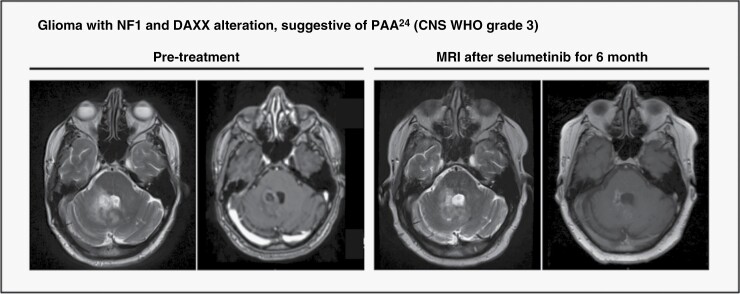
Case example of a male patient with a Glioma with NF-1 and DAXX alteration, suggestive of PAA.^24^ Baseline MRI scan (T2 weighted and contrast-enhanced T1 weighted sequences) at progression after CCNU alkylating chemotherapy and 6-month follow-up MRI scan (T2 weighted and contrast-enhanced T1 weighted sequences) following MEKi Selumetinib revealing partial response.

A 62-year-old male patient showed a T2-hyperintense contrast enhancing lesion in the left cerebellar hemisphere. He underwent stereotactic biopsy and was diagnosed with IDH-wild-type, MGMT non-methylated glioma CNS WHO grade 3 (suggestive of PAA^24^) and was treated with radiochemotherapy with adjuvant temozolomide (TMZ). After 2 cycles of adjuvant temozolomide, the patient presented with clinical deterioration, dizziness, and ataxia. A subsequent MRI as well as a confirmatory FET-PET scan was indicative of PD. Consequently, Lomustin (CCNU) was initiated and NGS-analysis was performed after interdisciplinary discussion in our neuro-oncology tumor board. NGS analysis revealed a molecular alteration within the Death-associated protein 6 (DAXX) with a truncating NF1 mutation. Based on clinical data of NF1-associated glioma, the MTB recommended treatment with the MEK-inhibitor (MEKi) Selumetinib (ESCAT IIIA).^25,26^ Treatment with selumetinib was initiated after further clinical deterioration and progression on an MRI scan performed after 2 cycles of CCNU. Follow-up MRI 6 months after the initiation of selumetinib showed regression of cerebellar tumor manifestation (Figure 4).

## Discussion

Targeted therapies are undoubtedly an evolving field and progress made in sequencing technologies offers the potential to identify and establish drivers in different cancer entities.

We here present our institutional experience of implementing precision oncology strategies in the framework of a MTB for glioma patients between January 2020 and June 2021. Our retrospective study demonstrates feasibility of detecting clinically relevant somatic alterations in selected, heavily pretreated glioma patients to guide clinical trial enrollment and identification of investigational or off-label drug opportunities. Simultaneously, we could show that molecularly matched targeted therapy for glioma patients is well tolerated and associated with prolonged disease control when compared to previous treatment modalities in certain patients. Within our cohort of 73 recurrent glioma patients, we observed actionable mutations in 68.5% of the cases and targeted therapy was administered in 24% of these patients, which is in line with previous studies for neuro-oncologic patients.^15,16,23^ However, in comparison to other solid tumor entities like pancreatic cancer (26%)^27^ or extrahepatic cholangiocarcinoma (40%)^28^ we detected a higher number of actionable targets highlighting the great variety of molecular-based treatment options for glioma patients. However, the assessment of the clinical utility of targeted therapies is not easy to define as molecular-based targeted therapies are mainly used at various stages of the disease. Therefore, 2 groups were generated: One group comprising all patients showing PD at first follow-up MRI and another group with at least stable disease. In addition, we calculated the PFS ratio as an intra-individual outcome parameter in order to avoid the comparison of patients with different neuro-oncologic diseases. In line with other studies which investigated the clinical utility of targeted therapies in CNS cancer patients,^23^ we observe a PFS ratio >1.3 in 25% (2/8) of our patients. Prospective clinical trials on targeted therapy in non-CNS cancer patients report on a PFS ratio >1.3 in 22.4% to 35%^21,22,29^ of the treated patients underlining that a subset of recurrent glioma patients may indeed benefit from a molecular-based targeted therapy. However, with the growing importance of personalized treatment strategies current outcome parameters need to be refined. The recently published EANO guidelines for molecular testing in glioma recommend the ESMO Magnitude of Clinical Benefit Scale as a potential tool.^30,31^ A group from Tuebingen further refined the grading system by considering also RANO criteria. Renovanz et al. therefore introduced the Neuro-Oncology Magnitude of Clinical Benefit Scale (Neuro-MCBS) comprising four grades, as a modification of the ESMO Magnitude in Clinical Benefit Scale (ESMO-MCBS).^23^ However, further studies in larger cohorts are urgently needed to validate this scale for routine use in neuro-oncologic patients.

In the past, several studies also investigated feasibility and significance of molecular-guided targeted therapy in neuro-oncology. However, these studies were mainly carried out in small patients cohorts and also include patients who underwent advanced molecular testing for first-line treatment.^32,33^ For example, Blumenthal et al. reviewed the clinical utility and response rates in correlation to NGS results of 34 glioblastoma patients of which no response was observed highlighting the need of further studies in larger cohorts on resistance mechanisms in order to implement molecular-based targeted therapy in neuro-oncology.^34^

With the advent of precision medicine managing of tumor patients became increasingly depended on individualized treatment strategies based on tumor sequencing data. However, not all molecular alterations within a tumor have equal biological consequences. Therefore, interpretation of NGS requires elucidating whether the observed variants really alter the wild-type function. Given that, understanding of the functional relevance of a molecular alteration and translation into the most appropriate therapeutic decision is complex and should be pursued within the framework of an interdisciplinary MTB.

Considering the high diagnostic effort, the therapeutic significance of molecular-matched therapy must be subject to critical assessment especially in a recurrent situation since intratumoral heterogeneity, subclonal expression of antigens and microenvironmental interactions remain a tough competitors in the therapeutic management.^35,36^ We also need to consider that advanced molecular diagnostic was done in heavily pretreated patients leaving a great deal of uncertainty whether the respective target still exists at the time of treatment, especially if primary tumor tissue was used for molecular diagnostics. Apart from the fact that testing at the time of first diagnosis might be meaningful if no standard of care exists for the respective entity or a potential promising treatment for a target in that entity is likely, where upfront treatment could be considered, we tried to use tissue samples that were collected after the last line of therapy, in order to provide the most accurate analysis of molecular profile data which was successful in 95% (69/73).

However, there are different perspectives on the genomic evolution of glioma during the course of disease. Kim et al. postulate that the mutational landscape of recurrent glioma only has little overlap with the primary tumor while the GLASS Consortium could show that the clonal architecture of each tumor remains similar over time leaving little evidence of recurrence-specific gene patterns.^35,37^ This being said, future studies have to consider both prevalence and persistence of distinct molecular alterations in primary and recurrent tumor tissue capturing the evolution of every individual tumor.

Nevertheless, we observed some particularly well-performing drugs in our cohort eg, the combination of Trametinib/Everolimus (ESCAT IID). Trametinib acts as an a *BRAF*/mitogen-activated protein kinase kinase (*MEK*) inhibitor and is approved for the adjuvant treatment of melanoma with BRAF^V600E^ or ^−V600K^ mutations. Everolimus is an inhibitor of the mammalian target of rapamycin (mTOR) playing a central role in key cellular processes, including cell cycle progression, protein synthesis, angiogenesis, and apoptosis and autophagy. Additionally, it proved efficacy in recurrent pediatric low-grade glioma.^38–40^ A combinatorial approach was administered in a patient with diffuse leptpomeningeal glioneuronal tumor and KIAA1549-BRAF fusion who shows durable response over more than 11 months. The MEK-inhibitor Selumetinib was recommended in a glioma patient with NF1 mutation based on preclinical and clinical study data showing durable response in pediatric low-grade glioma (ESCAT IIIA) leading to an ongoing remission over almost 1 year.^26,41,42^ An IDH mutation was the most common alteration within our cohort. Based on studies showing clinical and molecular remissions in patients with acute myeloid leukemia and several case studies in IDH-mutant advanced glioma the small molecule Ivosidenib was recommended for certain patients (ESCAT IIA). However, we observed variable response patterns with prolonged PFS, notably in low-grade, non-contrast-enhancing tumors, consistent with data published by Mellinghoff et al.^43^

Since standard first-line treatment has been defined for various brain tumor entities, guidelines in the recurrent situation are mostly lacking. In the absence of on-label treatment options, patients must be matched with actual clinical trials or off-label treatment options based on the respective molecular profile. A prime example in which the molecular signature of the tumor guides enrollment in the treatment arm is the NCT neuro master match (N2M2; NOA20).^44^ Another innovative clinical trial concept is GBM AGILE (Adaptive Global Innovative Learning Environment for Glioblastoma). Guided by a Master Protocol, GBM AGILE allows multiple drugs to be evaluated simultaneously and/or overtime against a common control for newly diagnosed and recurrent glioblastoma.^45^ A Master protocol and an Adaptive platform trial (APT)-design is also used for INSIGhT (Individualized Screening Trial of Innovative Glioblastoma Therapy). Multiple experimental arms are being compared with a common control of standard radiochemotherapy with temozolomide followed by adjuvant chemotherapy and can be enlarged or terminated according to their probability of success.

However, failure to address the individual molecular signature or the inability to perform the analysis in a clinically acceptable time frame can impair the outcome of individual patients. In our study 2, patients with requested and approved molecular-matched targeted therapy experienced rapid clinical deterioration within the timeframe of initiation of the molecular diagnostic to treatment initiation resulting in a subsequent change of the therapeutic objective. Consequently, the optimal timepoint for NGS analysis is one of the most debated topics in precision oncology for neuro-oncologic patients.^46^ Furthermore, functional and clinical interpretation of the sequencing data comprising prioritization of putative actionable alterations and matching of individual patients with the specific portfolio of investigational therapies and clinical trials, which undergo continuous revisions, often relies on manual procedures leading to considerable challenges for medical professionals. This fact, along with the objective to accelerate the latency period, underlines the need for standardization of the MTB strategy. First efforts were done by Tamborero et al. by developing a clinical decision support system to tackle these challenges through efficient data analysis.^7^ However, further studies are warranted to further optimize the processes towards patient-tailored treatment recommendations.

With the advent of the precision medicine era tumor agnostic treatments selective for specific molecular alterations are on the rise. In our retrospective analysis of recurrent glioma patients, we could shed light on therapeutic significance of molecular-guided targeted therapy in recurrent glioma patients. Our data might serve as a reasonable rationale for possible prospective controlled trials of molecularly stratified glioblastoma patients.

## Supplementary Material

vdad060_suppl_Supplementary_Figures

vdad060_suppl_Supplementary_Figure_Legends
