# Supplementary figures and images for: Significance of molecular diagnostics for therapeutic decision-making in recurrent glioma
Source: Neurooncol Adv. 2023 May 12;5(1):vdad060. doi: 10.1093/noajnl/vdad060 (PMC10243988; doi:10.1093/noajnl/vdad060)

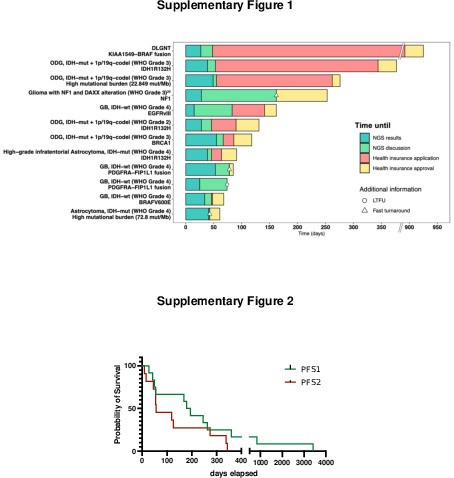

Supplement: vdad060_suppl_Supplementary_Figures [file vdad060_suppl_supplementary_figures.jpeg]
