# Supplementary material for: Significance of molecular diagnostics for therapeutic decision-making in recurrent glioma
Source: Neurooncol Adv. 2023 May 12;5(1):vdad060. doi: 10.1093/noajnl/vdad060 (PMC10243988; doi:10.1093/noajnl/vdad060)
Supplement: vdad060_suppl_Supplementary_Figure_Legends [file vdad060_suppl_supplementary_figure_legends.docx]

**Supplementary Figure 1**: Swimmer plot depicting distinct turnaround times from NGS request until MTB treatment implementation.

**Supplementary Figure 2**: Kaplan-Meier-Plot showing PFS of the MTB treatment recommendation (PFS2) and PFS of the last treatment modality (PFS1).
